# Supplementary material for: A Novel System for the Detection of Spontaneous Abortion-Causing Aneuploidy and Its Erroneous Chromosome Origins through the Combination of Low-Pass Copy Number Variation Sequencing and NGS-Based STR Tests
Source: J Clin Med. 2023 Feb 23;12(5):1809. doi: 10.3390/jcm12051809 (PMC10003649; doi:10.3390/jcm12051809)
Supplement: Supplementary file 1 [file jcm-12-01809-s001.zip › jcm-2176626-supplementary.pdf]

Table S1. Percentage of STR test results on all chromosomes of No. 4 miscarriage sample

**Chr**	**Num**	**M1M1M1**	**M1M1M2**	**M1M1P1**	**M1M2P1**	**P1P1P1**	**P1P1P2**	**P1P1M1**	**P1P2M1**	**MMM**	**MMP**	**PPP**	**PPM**
Chr1	26	15%	15%	69%	31%	15%	27%	50%	31%	31%	77%	31%	65%
Chr2	19	32%	5%	68%	21%	32%	26%	63%	21%	37%	74%	42%	74%
Chr3	19	21%	26%	68%	37%	21%	32%	58%	21%	37%	79%	47%	74%
Chr4	18	33%	33%	61%	28%	33%	17%	78%	22%	56%	72%	17%	83%
Chr5	15	40%	33%	93%	33%	40%	20%	47%	27%	53%	93%	27%	47%
Chr6	19	26%	26%	84%	42%	26%	37%	53%	32%	47%	89%	47%	68%
Chr7	14	21%	21%	64%	57%	21%	43%	64%	36%	29%	64%	43%	79%
Chr8	12	50%	50%	92%	50%	50%	33%	67%	42%	67%	100%	67%	83%
Chr9	13	31%	38%	69%	46%	31%	23%	77%	23%	54%	77%	46%	77%
Chr10	15	47%	13%	93%	33%	47%	40%	47%	47%	60%	100%	67%	73%
Chr11	16	31%	31%	75%	44%	31%	25%	62%	38%	50%	88%	25%	62%
Chr12	14	14%	36%	71%	71%	14%	36%	50%	36%	43%	93%	43%	79%
**Chr13**	7	0%	0%	43%	57%	0%	43%	14%	**100%**	0%	57%	43%	**100%**
Chr14	12	17%	42%	58%	50%	17%	42%	67%	50%	50%	83%	42%	75%
Chr15	9	56%	22%	78%	22%	56%	33%	78%	33%	56%	78%	56%	78%
Chr16	11	18%	27%	55%	36%	18%	45%	64%	36%	36%	73%	45%	73%
Chr17	9	56%	44%	78%	33%	56%	33%	78%	44%	78%	89%	44%	78%
Chr18	19	42%	47%	74%	32%	42%	11%	68%	16%	74%	89%	26%	68%
Chr19	4	0%	50%	75%	50%	0%	75%	75%	75%	50%	75%	75%	100%
Chr20	8	38%	25%	75%	25%	38%	25%	62%	25%	50%	75%	38%	75%
**Chr21**	6	0%	33%	83%	50%	0%	17%	17%	33%	33%	**100%**	17%	33%
Chr22	8	12%	38%	38%	25%	12%	0%	75%	25%	50%	62%	0%	75%
ChrX	11	100%	36%	73%	36%	73%	73%	73%	73%	100%	73%	73%	73%

Table S2. Trisomy

	M1M2P1	M1M1P1	MMP	P1P2M1	P1P1M1	PPM
**Trisomy**	70	18	73	4	4	0
**Total**		161			8	
**Ratio**		94.7%			5.3%	

*The number in the table means counted numbers.

Table S3. Triploid

	MMP	PPM
**Triploid**	31	9
**Total**	40	
**Ratio**	77.8%	22.2%

*The number in the table means counted numbers.

Table S4. Monosomy

	M	P
**X-monosomy**	16	13
**21-momosomy**	0	3
**Total for each**	16	16
**Total for all**	32	
**Ratio**	50%	50%

*The number in the table means counted numbers.

Table S5. UPD

	M1M1	P1P1
**UPD**	1	9
**11UPD**	0	4
**18UPD**	2	0
**Total for each**	3	13
**Total**	16	
**Ratio**	19.00%	81.00%

*The number in the table means counted numbers.

Table S6. Double trisomy

No.	M1M2P1 Count(chr#)	M1M1P1 Count(chr#)	MMP Count(chr#)	P1P2M1 Count(chr#)	P1P1M1 Count(chr#)	PPM Count(chr#)
**1**	/	/	2(14, 16)	/	/	/
**2**	/	/	/	/	2(8, 14)	/
**3**	/	1(22)	/	/	1(21)	/
**4**	/	/	1(21)	1(13)		/
**5**	/	1(18)	1(22)	/	/	/
**6**	/	/	2(15, 18)	/	/	/
